# The Brain Response to Peripheral Insulin Declines with Age: A Contribution of the Blood-Brain Barrier?

**DOI:** 10.1371/journal.pone.0126804

**Published:** 2015-05-12

**Authors:** Tina Sartorius, Andreas Peter, Martin Heni, Walter Maetzler, Andreas Fritsche, Hans-Ulrich Häring, Anita M. Hennige

**Affiliations:** 1 Department of Internal Medicine, Division of Endocrinology, Diabetology, Vascular Disease, Nephrology and Clinical Chemistry, University of Tuebingen, Tuebingen, Germany; 2 German Center for Diabetes Research (DZD), Tuebingen, Germany; 3 Institute for Diabetes Research and Metabolic Diseases of the Helmholtz Center Munich at the University of Tuebingen (IDM), Tuebingen, Germany; 4 Department of Neurodegenerative Diseases and Hertie Institute for Clinical Brain Research, University of Tuebingen, Tuebingen, Germany; 5 German Center for Neurodegenerative Diseases (DZNE), Tuebingen, Germany; Hungarian Academy of Sciences, HUNGARY

## Abstract

**Objectives:**

It is a matter of debate whether impaired insulin action originates from a defect at the neural level or impaired transport of the hormone into the brain. In this study, we aimed to investigate the effect of aging on insulin concentrations in the periphery and the central nervous system as well as its impact on insulin-dependent brain activity.

**Methods:**

Insulin, glucose and albumin concentrations were determined in 160 paired human serum and cerebrospinal fluid (CSF) samples. Additionally, insulin was applied in young and aged mice by subcutaneous injection or intracerebroventricularly to circumvent the blood-brain barrier. Insulin action and cortical activity were assessed by Western blotting and electrocorticography radiotelemetric measurements.

**Results:**

In humans, CSF glucose and insulin concentrations were tightly correlated with the respective serum/plasma concentrations. The CSF/serum ratio for insulin was reduced in older subjects while the CSF/serum ratio for albumin increased with age like for most other proteins. Western blot analysis in murine whole brain lysates revealed impaired phosphorylation of AKT (P-AKT) in aged mice following peripheral insulin stimulation whereas P-AKT was comparable to levels in young mice after intracerebroventricular insulin application. As readout for insulin action in the brain, insulin-mediated cortical brain activity instantly increased in young mice subcutaneously injected with insulin but was significantly reduced and delayed in aged mice during the treatment period. When insulin was applied intracerebroventricularly into aged animals, brain activity was readily improved.

**Conclusions:**

This study discloses age-dependent changes in insulin CSF/serum ratios in humans. In the elderly, cerebral insulin resistance might be partially attributed to an impaired transport of insulin into the central nervous system.

## Introduction

Insulin resistance is one hallmark of type 2 diabetes mellitus (T2DM). While impaired insulin action in peripheral tissues like skeletal muscle, liver and fat is well documented in a large number of studies and thought to be a predictor of alterations in glucose homeostasis, the causes and consequences of insulin resistance in the brain are far less understood [1;2]. Since insulin exerts direct effects on brain function which impacts on eating behavior, body weight regulation, peripheral glucose metabolism and cognitive function, more insight into this decisive phenomenon is especially important [3].

Recent research demonstrates insulin resistance at a neural level in both obese humans and animals [4;5]. The underlying mechanisms include mediators that are present in obesity, such as elevated levels of saturated free fatty acids [6–8]. In addition, genetic variants [9–12] and aging [13] contribute to impaired insulin action in the brain. Aging is one of the main risk factors for the development of T2DM, and age-related modifications in central insulin action, including changes in the insulin concentration itself or its intracellular signaling pathways seem to be pronounced in neurodegenerative diseases such as Alzheimer’s disease (AD) [14]. However, although defective brain insulin signaling is considered as an important feature of AD pathology, the underlying mechanisms of reduced insulin signaling in AD remain unknown.

We previously reported an insulin-mediated increase in cortical activity in lean mice, whereas high-fat diet fed mice displayed insulin resistance in the brain [4]. A number of human magnetoencephalography (MEG) studies revealed that a profound increase in cortical activity is ascribed to a rise in insulin concentrations within the brain [7;9]. By using functional magnetic resonance imaging techniques (fMRI) insulin’s impact on certain areas in the brain that represent the regulation of metabolism, object processing, memory and locomotion was demonstrated [15–17]. Following intravenous insulin injection in a hyperinsulinemic-euglycemic clamp, the insulin response on brain activity declined with age; however, the underlying mechanisms remained obscure [13;18].

Besides a direct effect at the level of the insulin receptor in neuronal cells, alterations in insulin transport from the periphery into the central nervous system might account for diminished insulin action. Rodent studies with radiolabeled insulin demonstrated that the transport of insulin across the BBB is saturable already by low doses of insulin, and that additional raise in serum concentrations will not be reflected in proportionate increases in the levels in the CNS [19;20]. Former studies provided evidence that aging animals and humans have altered blood-brain barrier (BBB) function of carrier-mediated transport systems [21]. In particular, in the pathophysiology of neurodegenerative disorders, modifications at the BBB are supposed to account for alterations in cognitive function and even dementia [22–24].

Research in animals clearly indicated that insulin from the blood enters the brain via a saturable receptor-mediated transport [25], and obesity was shown to decrease transport of insulin into the cerebrospinal fluid (CSF) in rats [26], while data in humans are rare. Although the majority of studies suggest that the major quantity of insulin crosses non-brain capillary endothelial barriers and the BBB by receptor-mediated transport [27;28], one has to take into account that insulin has also been shown to cross the BBB by different mechanisms, like extracellular pathways, non-saturable transmembrane diffusion or saturable transport [29]. Of interest, it was shown that the number of insulin receptors is down-regulated in the presence of high insulin concentrations in cultured endothelial cells, and that the transport rate for insulin is reduced in proportion to the reduction in receptor number [30]. Furthermore, one recent study suggested impaired transport in insulin resistant persons [31]. Additionally, it was demonstrated that high saturated fat and high glycemic diet lowered CSF insulin concentrations in healthy individuals [32], and impaired insulin sensitivity has been linked to cognitive failures and structural and functional brain deficits in the elderly [33]. Insulin signaling appears to play a key role in regulating lifespan and inadequate insulin action may limit glucose utilization, synaptic plasticity, and survival signaling in Alzheimer’s disease [34;35]. Moreover, a decrease of insulin sensitivity with aging has been linked to a reduction in hepatic parasympathetic nervous activity which plays a crucial role in the postprandial state [36].

It has still to be investigated whether impaired insulin action in the CNS may be a direct consequence of insulin transport into the brain across the BBB or if neuronal insulin resistance may account for this impairment. In an exploratory study, we measured age-dependent changes of serum and CSF insulin concentrations in human subjects. To complement these data, we experimentally addressed insulin action in the context of aging. Therefore, an animal model was used and the impact of insulin on cortical activity was assessed. Insulin concentrations were enhanced in young and aged mice by both subcutaneous (s.c.) injection, that requires crossing the BBB to elicit brain effects, and by intracerebroventricular (i.c.v.) injection, that bypass the BBB. Our study discloses age-dependent changes of CSF/serum ratios of insulin in humans. Moreover, using mice of different age bracket, we demonstrated an impaired insulin action in the CNS after peripheral s.c. insulin injection in aged mice, while insulin action after direct central i.c.v. applied insulin is still intact in the aged brain of these mice. These data suggest that in the elderly, cerebral insulin resistance might be partially attributed to an impaired transport of insulin into the CNS which impacts neuronal brain activity.

## Materials and Methods

### Analytical procedures in humans

Paired serum and CSF samples from 160 human subjects (61 male, 99 female, age 55.7±1.5 years±SEM were included in the exploratory study. NaF-Plasma was also available in 140 subjects for glucose measurements. The subjects underwent lumbar punctures for diagnostic reasons in the morning prior to breakfast and had no signs of deteriorated blood-CSF barrier or intrathecal IgG production, intra-cerebral hemorrhage, or increased CSF cell count. Individuals with blood glucose levels above 200 mg/dL were excluded from the study. Patient records and information was anonymized and de-identified prior to analysis. The ethics committee of the University of Tuebingen approved the study. The study was performed in accordance with the Declaration of Helsinki. Plasma and CSF glucose was measured immediately after collection on the ADVIA 1800 clinical chemistry analyzer (hexokinase method) and CSF and serum IgG and albumin concentrations were measured using the BN ProSpec nephelometer. Insulin levels were promptly assessed after blood and CSF collection and determined on the ADVIA Centaur chemiluminescent immunoassay system. The assay was tested for linearity and recovery for insulin in CSF and the variation in CSF at an insulin concentration of 8 pmol/L was 12% (all Instruments from Siemens Healthcare Diagnostics, Eschborn, Germany).

### Animals

C57BL/6NCrl male mice at the age of 10–12 weeks (young) and 77–95 weeks (aged) (from Charles River Laboratories) were used. They were maintained on 12:12 light/dark conditions (lights on from 7 a.m. to 7 p.m.) and had free access to water and chow. All animal procedures were conducted according to the guidelines of laboratory animal care and were approved by the Committee on the Ethics of Animal Experiments of the University of Tuebingen and the Regional Board Tuebingen (Permit Number: M1/06) for animal research. Surgeries were performed under ketamine/ xylazine and isoflurane anesthesia and animals got all efforts to minimize suffering.

### Insulin treatment and metabolic characterization in mice

Mice received a subcutaneous injection of insulin glargine (lantus, 1 unit/kg body weight) or vehicle solution (0.9% sterile NaCl) 2 h before the night-active phase (5 p.m.) for four consecutive days in a crossover design 5 days apart. Glucose levels were sampled from mouse tail bleeds at 9 a.m. and 5 p.m. using a Glucometer Elite (Bayer Health Care, Leverkusen, Germany). To exclude inter-individual variation in cortical ECoG activity, the data for the ECoG power density are expressed as percentage change from vehicle application. An intracerebroventricular (i.c.v.) injection with human insulin (actrapid, Novo Nordisk, Denmark) in a concentration of 3.75 mU/ 5 μL (which correspond to 5.41 nmol/mL) was performed according to published literature [37–39] and as previously described [4].

### Electrocorticography (ECoG) telemetry transmitter implantation in mice

Methods used were similar to those described previously [6]. Anaesthetized young and old animals were placed in a stereotaxic head holder for the subcutaneous implantation of the telemetry ECoG transmitter. Two ECoG lead wires were connected to screws placed epidurally 1 mm anterior to lambda and 1 mm left to the sutura sagittalis for the recording electrode, and 1 mm anterior to bregma and 1 mm right to the sutura coronaria for the reference electrode. Therefore, these epidurally located conductions predominantly assess electrical activity from the cerebral cortex, more specified from pyramidal cells as excitatory projection neurons in the cerebral cortex, which are under influence of interactive subcortical structures. An intracerebroventricular cannula was additionally implanted in the left ventricle. Electrodes and screws were then fixed in place with dental acrylic cement. After a 1-week recovery period, telemetry signals (ECoG) were recorded and processed by a Data-Sciences analogue converter (Data Exchange Matrix, DSI) and stored digitally using the Dataquest A.R.T. 4.2 software (DSI).

### Western Blot analysis

A bolus of human insulin (Novo Nordisk, Bagsværd, Denmark) or a comparable amount of saline was intravenously (i.v.) injected into the inferior vena cava (1 U/ mouse for 10 min) or intracerebroventricularly (i.c.v.) in the lateral ventricle (3.75 mU/ 5 μL for 10 min) of overnight fasted mice under ketamine/ xylazine anaesthesia [4;37–39]. After decapitation, total brain or different brain regions (hippocampus, cortex) were quickly removed and homogenized in lysis-buffer. In brain lysates, visualization of immunocomplexes was accomplished after gel electrophoresis and Western blot experiments using antibodies directed against phospho-AKT (Ser473) (Upstate, Charlottesville, USA) or total AKT (Cell Signaling, Millipore, Billerica, Ma, USA). The nonradioactive enhanced chemiluminescence system ECL was used. GAPDH (Cell Signaling, Millipore, Billerica, Ma, USA) served as loading control. Parallel Western blots were run to detect unphosphorylated AKT. Optical densitometry was performed using ImageJ software (freely available at http://rsb.info.nih.gov/ij/index.html) to compare bands in autoradiographs.

### Data analysis and statistics

#### Rodent data

The data analysis for ECoG measurements was performed using standard criteria in rodents [40] and as previously described [4]. The data for cortical ECoG activity are expressed as power change (percentage of control (vehicle application)). If not stated otherwise, data are expressed as mean±SEM. Differences between animal groups were analyzed by two-tailed unpaired Student’s *t* test or one-way ANOVA (quantification of ECoG for the 4 day-measurement period); *P*<0.05 was considered significant.

#### Human data

Data that were not normally distributed (Shapiro-Wilk *W* test) were logarithmically transformed prior to statistical analysis. Correlations were analyzed using linear regression models. Results with *P*<0.05 were considered statistically significant. For all statistical analyses, the software package JMP10 (SAS Institute, Cary, NC, USA) was used.

## Results

### CSF/serum ratio for insulin is reduced in aging subjects

**Fig 1:**
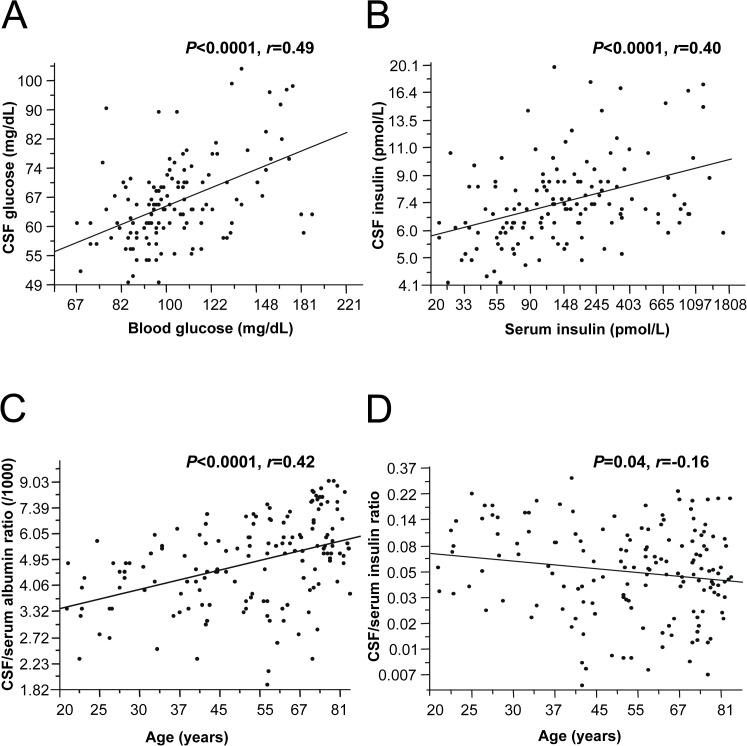
Concentrations of glucose, insulin and albumin in human paired CSF/serum samples. A: Correlation of CSF glucose with plasma glucose concentrations in humans (*P*<0.0001, *r* = 0.49). B: Correlation of CSF insulin with serum insulin concentrations in humans (*P*<0.0001, *r* = 0.40). C: Increased CSF/serum ration for albumin as marker for the blood-CSF barrier in older people and strong positive correlation with age (*P*<0.0001, *r* = 0.42). D: CSF/serum ratio for insulin in older people and negative correlation with age in humans (*P* = 0.04, *r* = -0.16).

In an exploratory study, 160 human subjects (61 male, 99 female, age 55.7±1.5 years±SEM undergoing lumbar punctures for diagnostic reasons) were included. They had no signs of deteriorated blood-CSF barrier as assessed by the CSF/serum albumin ratio or intrathecal IgG production. CSF glucose concentrations were about 37% lower than blood glucose concentrations (66.2±0.86 mg/dL *vs*. 105.7±2.2 mg/dL). As expected, CSF glucose concentrations were closely correlated with plasma glucose concentrations (*P*<0.0001; *r* = 0.49) (Fig 1A). The average CSF insulin concentration was 8.2±0.2 pmol/L with 95% confidence interval ranging from 5.0 to 13.7 pmol/L. On average, the CSF insulin concentration was 8.0±0.5% of the serum value. Serum and CSF insulin concentrations were tightly correlated (*P*<0.0001; *r* = 0.40) (Fig 1B).

The blood-CSF barrier generally gets more permeable with age leading to increased CSF albumin concentrations and a higher CSF/serum albumin ratio in older subjects [41]. This was also detectable in our set of samples (*P*<0.0001; *r* = 0.42) (Fig 1C). When we next investigated whether the ratio for insulin is also age-dependent, we detected a negative correlation of the insulin CSF/serum ratio with age (*P* = 0.04; *r* = -0.16) (Fig 1D), suggesting that the blood-CSF barrier is less permeable for this hormone in aged subjects.

### Phosphorylation of AKT in the brain is diminished in aged mice

**Fig 2:**
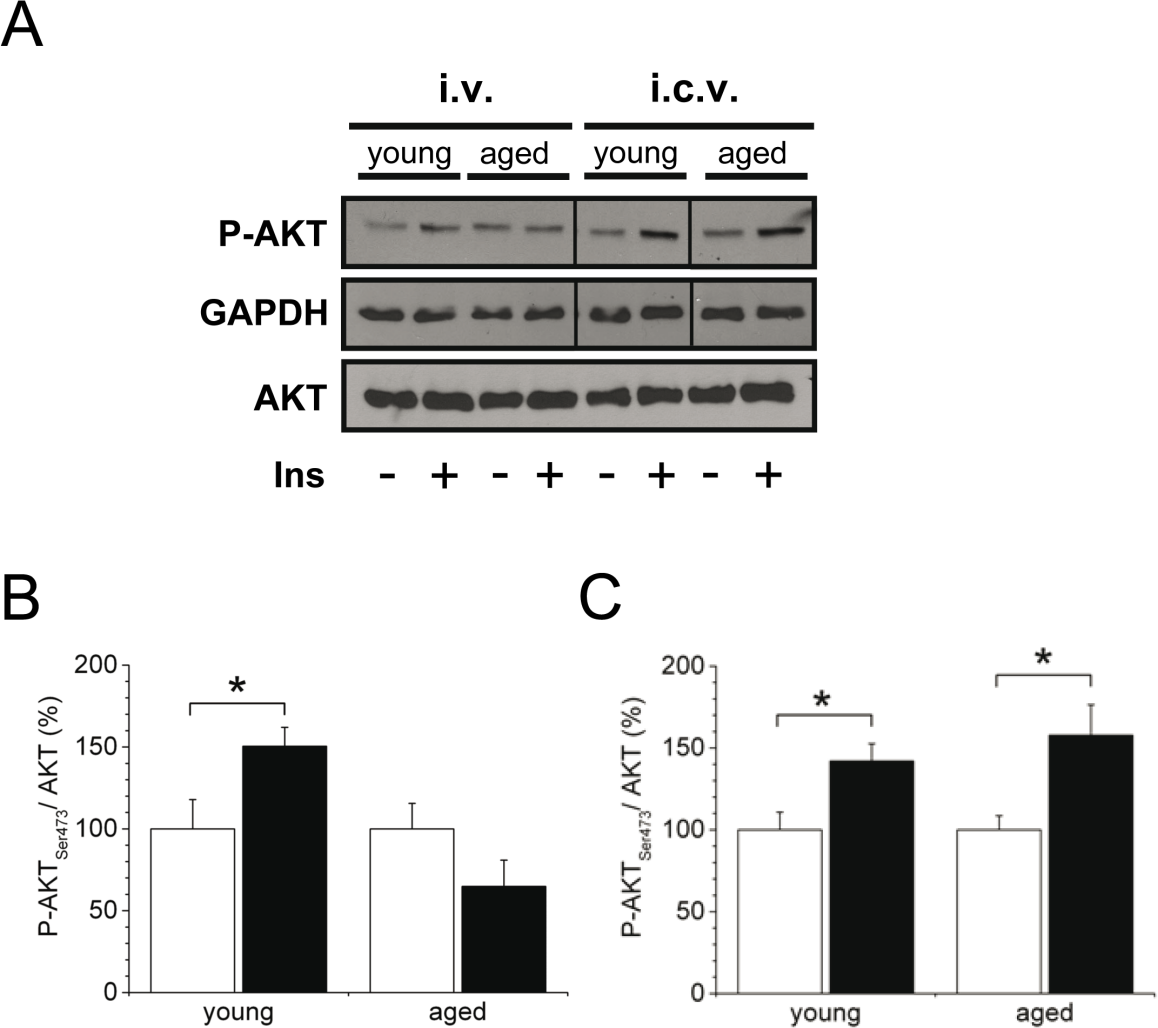
Insulin signaling in the brain in young and aged mice after intravenous or intracerebroventricular insulin stimulation. A-C: Western Blot analysis in brain tissues after intravenous (i.v.) or intracerebroventricular (i.c.v.) human insulin or vehicle injection in overnight fasted animals. A: Representative Western Blot out of 5 independent experiments of phospho-AKT (Ser473) (P-AKT) and AKT. Parallel Western blots were run to detect unphosphorylated AKT. B,C: Quantification of P-AKT after i.v. (B) or i.c.v. (C) injection of insulin (black) or vehicle (white). P-AKT was quantified based on scanning densitometry of western blots normalized for GAPDH, and the values are calculated by the relative density (P-AKT/AKT ratio). Ins, insulin. Data are mean ± SEM; *n* = 5–9 per condition and group; *P<0.05.

To dissect the pathways that are involved in age-dependent insulin action in the brain, we first investigated insulin signaling in whole brain lysates. When we applied a bolus insulin injection in the inferior vena cava and harvested brain tissues, Western blot analysis revealed that phosphorylation of the insulin signaling element AKT was lower after insulin-stimulation in aged mice compared with young animals (Fig 2A and 2B). Strikingly, phosphorylation of AKT in brain lysates of aged mice treated intracerebroventricularly with insulin was significantly enhanced and comparable to levels of young mice (Fig 2A and 2C). Similar results were found in individual brain regions like cortex and hippocampus of young and aged mice peripherally or centrally stimulated with insulin (S1 Fig). In accordance with the human data, these results further indicate that impaired insulin action in the brain originates from altered transport of the hormone into the brain with increasing age, while insulin action within the brain, particularly at the level of AKT, remains intact.

### No change of blood glucose and body weight in young and aged mice by subcutaneous insulin

**Fig 3:**
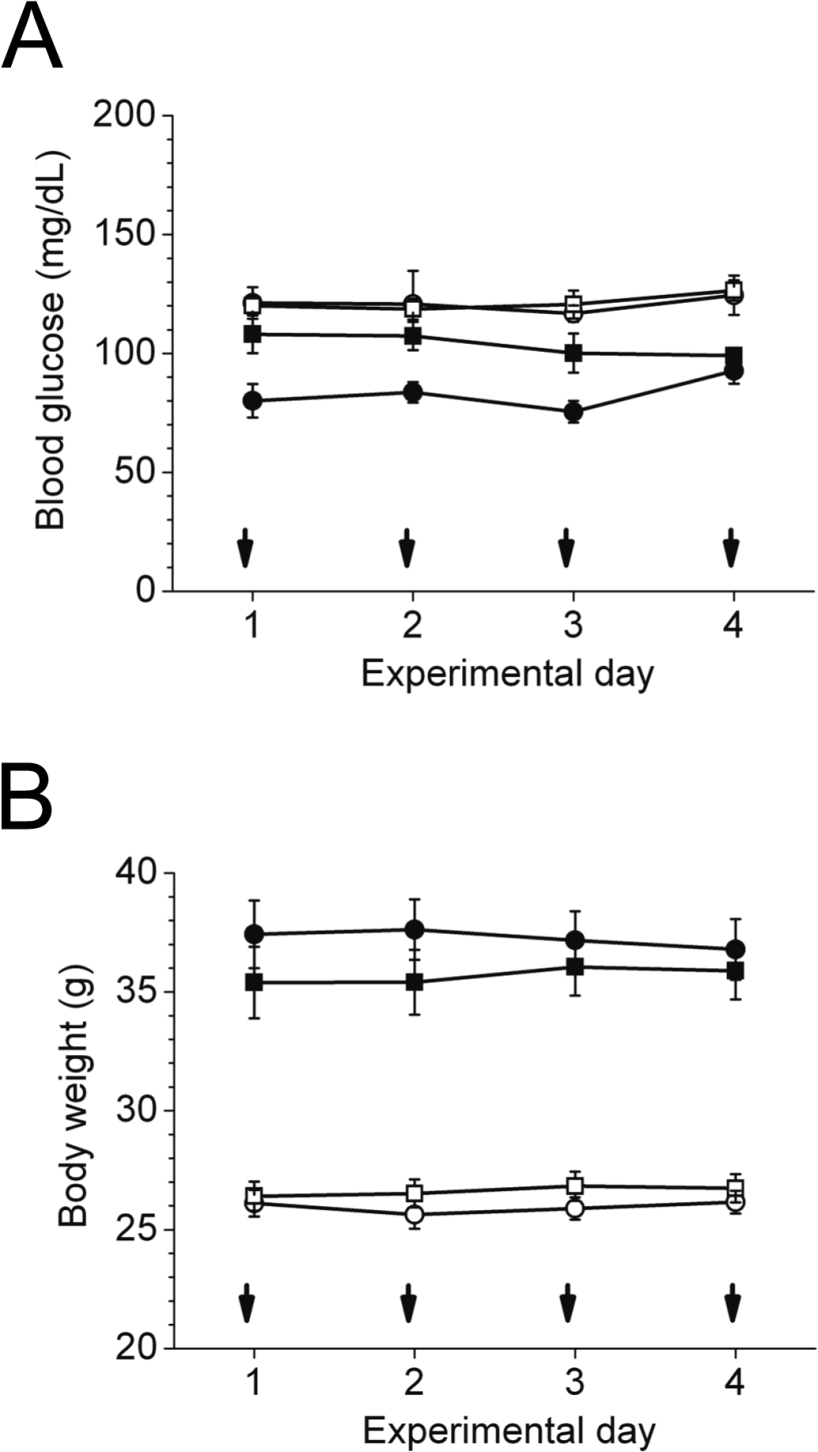
Blood glucose concentrations and body weight in young and aged mice during the 4-day insulin glargine treatment. Young (white symbols, *n* = 9–10) and aged (black symbols, *n* = 6) C57BL/6 mice were injected once daily (5 p.m.) with insulin glargine (circles) or vehicle (squares) for 4 consecutive days (arrow indicate s.c. injection time point). A,B: Blood glucose levels from tail bleeds (A) and body weight (B) at 5 p.m. Data are mean±SEM.

To complement previous results, we made use of mice that were subcutaneously treated with a long-acting insulin analogue to obtain sustained insulin action. Repeated subcutaneous injections were performed on four consecutive days. Blood glucose levels, assessed shortly before 5 p.m. injection, did not show any alteration due to treatment (Fig 3A). In addition, the averaged blood glucose concentration for the 4-day treatment period 16 hours after injection (9 a.m.) was not different between insulin and saline treated mice (young: 135.7±4.8 *vs*. 129.3±3.4 mg/dL, *P* = 0.28; aged: 96.3±4.4 *vs*. 86.6±3.1 mg/dL, *P* = 0.08). Measures of body weight displayed no change during the 4-day treatment (Fig 3B).

### Delayed increase of brain activity in aged mice during subcutaneous insulin treatment

**Fig 4:**
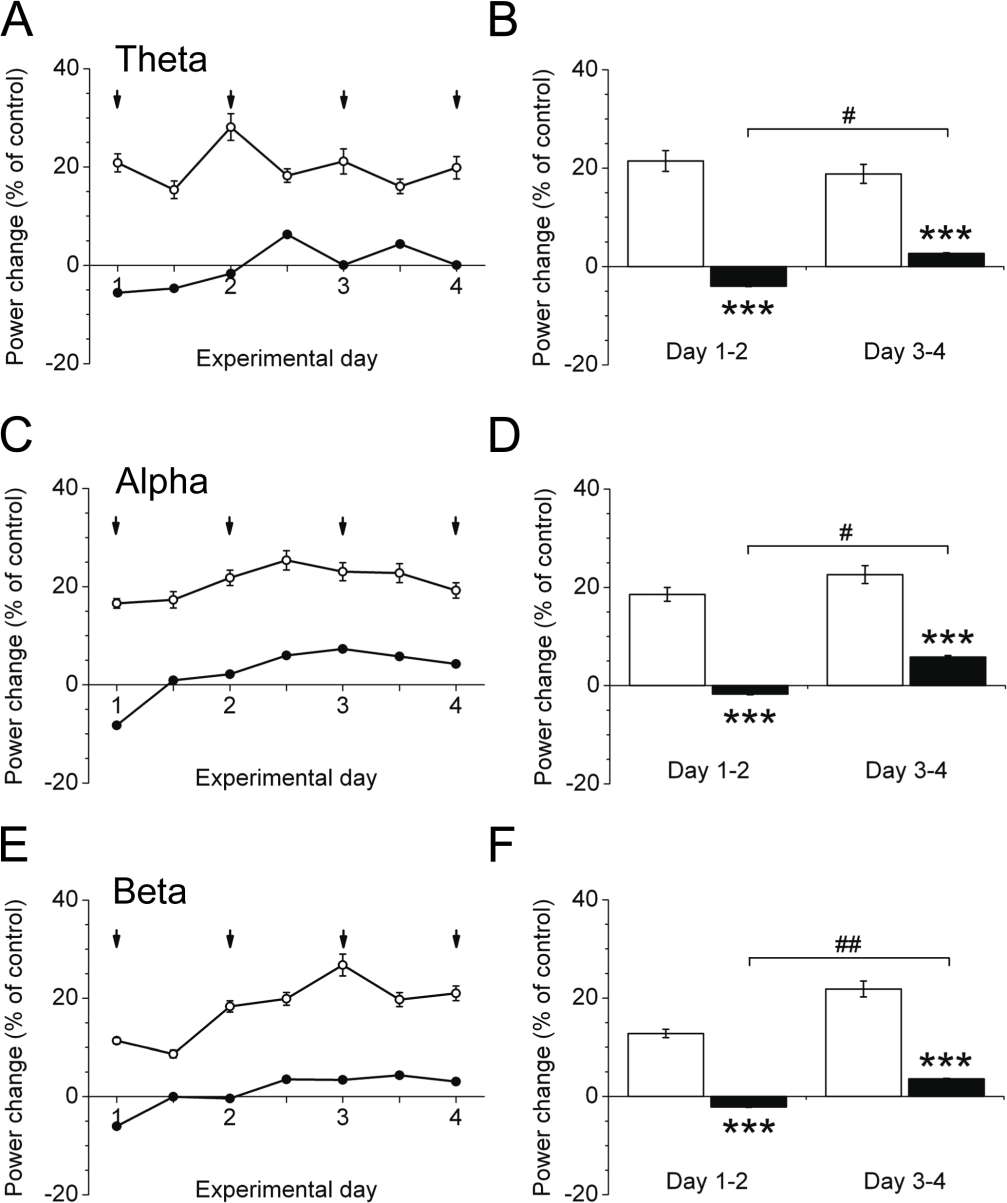
ECoG power spectral analysis in young (white) and aged (black) mice. Young (*n* = 9–10) and aged C57BL/6 mice (*n* = 6) were injected once daily (5 p.m.) with insulin glargine or vehicle for 4 consecutive days. A-F: ECoG power spectral analysis, calculated by fast fourier transformation (FFT) and expressed as % power change of control (vehicle application) during the 4-day lasting treatment period for the theta (4–8 Hz) (A,B), alpha (8–12 Hz) (C,D), and beta (12–30 Hz) (E,F) frequency bands. Data are indicated as time response during the insulin glargine treatment, denoted as 12-hour average±SEM (7 p.m. to 7 a.m. and 7 a.m. to 7 p.m.) (A,C,E) and as 2-day average±SEM (day 1–2, day 3–4) (B,D,F). Statistical significance to control injection is denoted as follows: ***P<0.001. Significance between the values under the brackets are ^#^ *P*<0.05, ^##^ *P*<0.005.

To follow up on the question whether insulin action in the brain depends on age as suggested by earlier studies in humans, young and aged mice were treated with subcutaneous insulin for four days. We choose cortical activity as readout for insulin action in the brain. To test whether subcutaneous insulin indeed alters measures of cortical activity, we performed brain analyses by using telemetric implants. Young mice treated with insulin displayed a significant increase in the insulin-sensitive theta (Fig 4A and 4B), alpha (Fig 4C and 4D) and beta (Fig 4E and 4F) frequency bands compared to vehicle application, while cortical activity in the delta frequency band was unchanged (data not shown). Of note, compared to young animals, aged mice reveal significantly reduced cortical activity for the total 4-day treatment period (Fig 4A, 4C and 4E). Interestingly, the response in cortical activity to insulin improved during the course of the 4 day treatment. However, aged mice still showed a significantly reduced insulin-mediated cortical activity compared to young animals after 4 days of insulin treatment (Fig 4B, 4D and 4F).

### Increased brain activity in aged mice after intracerebroventricular (i.c.v.) injection of insulin

**Fig 5:**
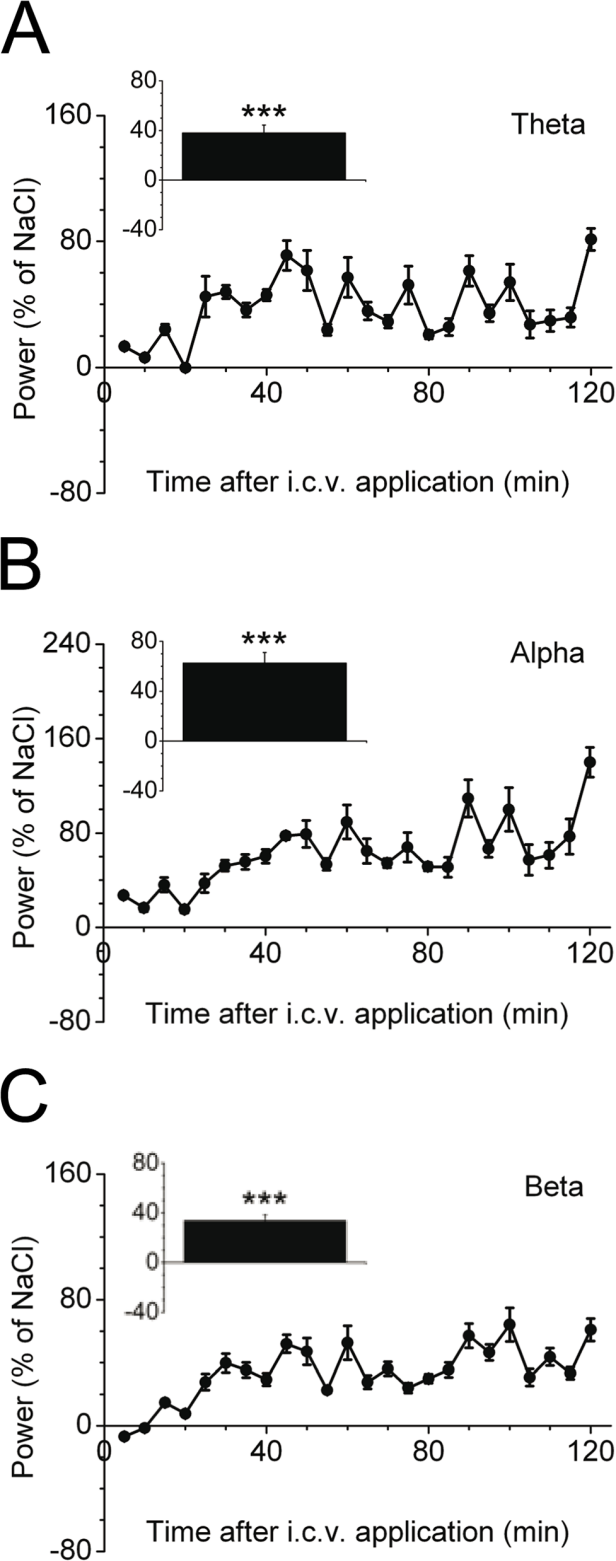
ECoG power spectral analysis in aged mice after intracerebroventricular (i.c.v.) injection of insulin. A-C: C57BL/6 mice (*n* = 6) were i.c.v. injected with human insulin or vehicle and cortical activity was calculated 120 min after the injection for (A) theta, (B) alpha, and (C) beta frequency bands. ECoG power spectral analysis is expressed as % power change of NaCl (vehicle application). Inserts illustrate the quantification of the averaged 120 min measurement period of respective frequency bands. Data are mean±SEM. Statistical significance is indicated as follows: ***P<0.001.

We further investigated the role of the blood-brain barrier in aging animals by analyzing cortical activity after an intracerebroventricular insulin stimulus in senescent mice. Insulin was able to readily increase cortical activity in the theta, alpha and beta frequency band (Fig 5A–5C) to the same extent as recently reported in young animals [4]. Thereby, the most prominent effect was seen in the alpha frequency during 2 hours post-injection (62.5 ± 8.5% compared to NaCl; *P*<0.001) (Fig 5B). This argues for an impaired transport of insulin into the brain to achieve a robust insulin-mediated increase in cortical activity in aged mice.

## Discussion

Insulin originates from pancreatic β-cells and is transported into the CNS across the blood-brain barrier (BBB) via a receptor-mediated, saturable process that is limited by the barrier system formed by tight junctions between endothelial cells [25;42]. Rodent studies with radiolabeled insulin demonstrated that this transport is saturable already by low doses of insulin, and that an additional raise in serum insulin levels will not be reflected in proportionate increases in CNS levels [19;20]. Adequate insulin action in the brain has been linked to a lean and healthy phenotype while insulin resistance in the brain is associated with obesity, glucose intolerance, physical inactivity and even Alzheimer´s disease [35;43;44]. A number of studies reported on insulin resistance in the human brain using MEG and fMRI measurement techniques [5;9;45–47]. As compared to insulin-resistant subjects, a decrease in body fat was shown to correlate with insulin sensitivity in the brain in a two year lifestyle intervention program [48]. However, it is still not fully understood to what extend defects at the neural level and impaired transport of the hormone into the brain account for impaired insulin action [25;49].

In general, the concentration of proteins that are not subject to a transport into the CSF is determined by two independent mechanisms: first the production of CSF in the ventricles and second the diffusion of proteins into the CSF on its way through the spinal channel. With increasing age, the production of CSF decreases and diminishes the CSF flow velocity on the way through the spinal channel. This allows proteins more time to defuse into the CSF and thereby increases CSF protein concentrations as well as the CSF/serum protein ratio [41]. In our set of data, this was true for albumin. However, for insulin, our current data point to the fact that insulin transport into the central nervous system is delayed or even impaired in aged individuals and might contribute to a decline in brain activity, as supported by the rodent model. However, the present human study has some limitations and is still exploratory: we cannot fully answer the question whether a linear relationship between serum and CSF insulin concentrations also exists at higher serum insulin concentrations, as there were not many subjects in this range. To answer this question, a dynamic study or CSF insulin measurements at different time points after peripheral injection of insulin or a glucose challenge in humans would be necessary. Furthermore, we cannot investigate the effects of other covariates of insulin transport into the CSF like obesity or insulin resistance, since these variables were not available in the present set of data. However, the applied analysis of the CSF/serum ratios considered the variability in peripheral and central insulin and glucose concentrations.

In particular, clamp studies in aged human subjects suggested an impaired insulin transport across the BBB in cerebral insulin resistance, whereupon the insulin-mediated increase in brain activity specifically declined with age [13]. However, we cannot rule out that other mediators that go along with aging affect insulin transport across the BBB [50]. Especially reduced insulin sensitivity goes along with reduced transport of insulin into the CSF [31]. Furthermore, elevated blood glucose or free fatty acids levels are known in older persons; however, these measures were not different between the groups studied here. As we were not able to directly assess insulin transport across the BBB in humans, we decided to use measures of insulin-mediated brain activity in mice after peripheral and direct intracerebroventricular (i.c.v.) insulin application as readout for insulin action in the brain in a cross-over design. In aged mice, i.c.v. insulin injections increased brain activity, most distinctive in the alpha frequency band, while peripheral insulin was less effective and even time-delayed. By contrast, young animals responded properly to both routes of application. Evidence is presented that brain activity oscillations in the alpha frequency band in particular reflect cognitive and memory performance. This is predominantly mediated by the basal forebrain system and affects thalamo-cortical and cortico-cortical processing [51]. As i.c.v. injected insulin improves aspects of memory function in rats [37], one might speculate that in our study, i.c.v. applied insulin predominantly acts on the abovementioned brain systems to preferentially increase alpha frequency. The absence of significant changes in the delta frequency band after i.c.v. applied insulin in aged mice might be resigned to the fact that healthy aging is accompanied by a widespread decrease in slow wave (delta) power [52]. The time-delay after peripheral insulin injections might be partially attributed to an impaired transport of insulin into the CNS with impacts on neuronal brain activity, but has to be confirmed in future studies that should also include additional insulin doses. Nonetheless, with age, other factors might contribute to impaired cortical activity after insulin administration. As the neuronal system ages, it undergoes a variety of morphological and biochemical changes that include neuronal loss, synaptic destruction, memory and cognitive impairment, a loss of glutamate receptors, a decrease in the abundance of acetylcholine, dopamine, and norepinephrine, and an increase in oxidative stress and inflammation [53].

In consideration of the short administration period of 4 days, we did not expect any change in body weight as substantiated by our data. Furthermore, the aim of the study was to not alter blood glucose concentration after repeated insulin administration as this might further impact on the transport of insulin into the brain. For the duration of the study, we therefore choose a dose, where glucose levels remained in the euglycemic range without changes in food intake and finally body weight. The integrated coordination of neuronal responses through the phosphoinositide 3-kinase (PI3K)/AKT pathway has significant functional impact, as downstream insulin signaling at this level was shown to be impaired in high caloric diet-induced insulin resistant mice [4], and this pathway was also ascribed to be impaired by aging [54]. However, in our study, the AKT pathway of the insulin signaling cascade seems to be indeed intact in the elderly as phosphorylation of AKT at Ser473 after an i.c.v. insulin injection was comparable to young animals, both in whole brain lysates and different brain regions like hippocampus and cortex. This points towards a preserved neuronal insulin response in the presence of impaired transport into the brain in aged mice and highlights the impact of the route of insulin application.

A number of studies in humans used intranasal insulin to circumvent the BBB. In a former MEG-study, for example, we reported that obese humans did not respond to intranasal insulin [5]. This favors the idea that in obesity, resistance at the cellular level causes impaired brain activity, which is distinct from aging-related mechanisms that are located at the transport level. This is in line with former studies that provided evidence for altered BBB function and carrier-mediated transport systems that are affected by aging in animals and humans, such as the transport of choline [55–57].

Together, our data suggest that the permeability of the blood-brain barrier for insulin is impaired in aged mice and humans while insulin action in the brain remains intact. Besides potential morphological and biochemical changes, which were not in the focus of the present study, an inadequate insulin transport into the central nervous system may contribute to impaired insulin action in elderly subjects, which finally harms glucose homeostasis and neuronal function. This information is crucial for more efficient preventive and therapeutic interventions like specific insulin sensitizing agents in an aging population.

## Supporting Information

S1 Fig Insulin signaling in different brain tissue of young and aged mice after intravenous or intracerebroventricular insulin stimulation. A-B: Western Blot analysis of phospho-AKT (Ser473) (P-AKT) and AKT in hippocampus (A) and cortex (B) after intravenous (i.v.) or intracerebroventricular (i.c.v.) human insulin or vehicle injection in overnight fasted young and aged animals. Parallel Western blots were run to detect unphosphorylated AKT. Ins, insulin.
(TIF)
