# Supplementary figures and images for: The Brain Response to Peripheral Insulin Declines with Age: A Contribution of the Blood-Brain Barrier?
Source: PLoS One. 2015 May 12;10(5):e0126804. doi: 10.1371/journal.pone.0126804 (PMC4429020; doi:10.1371/journal.pone.0126804)

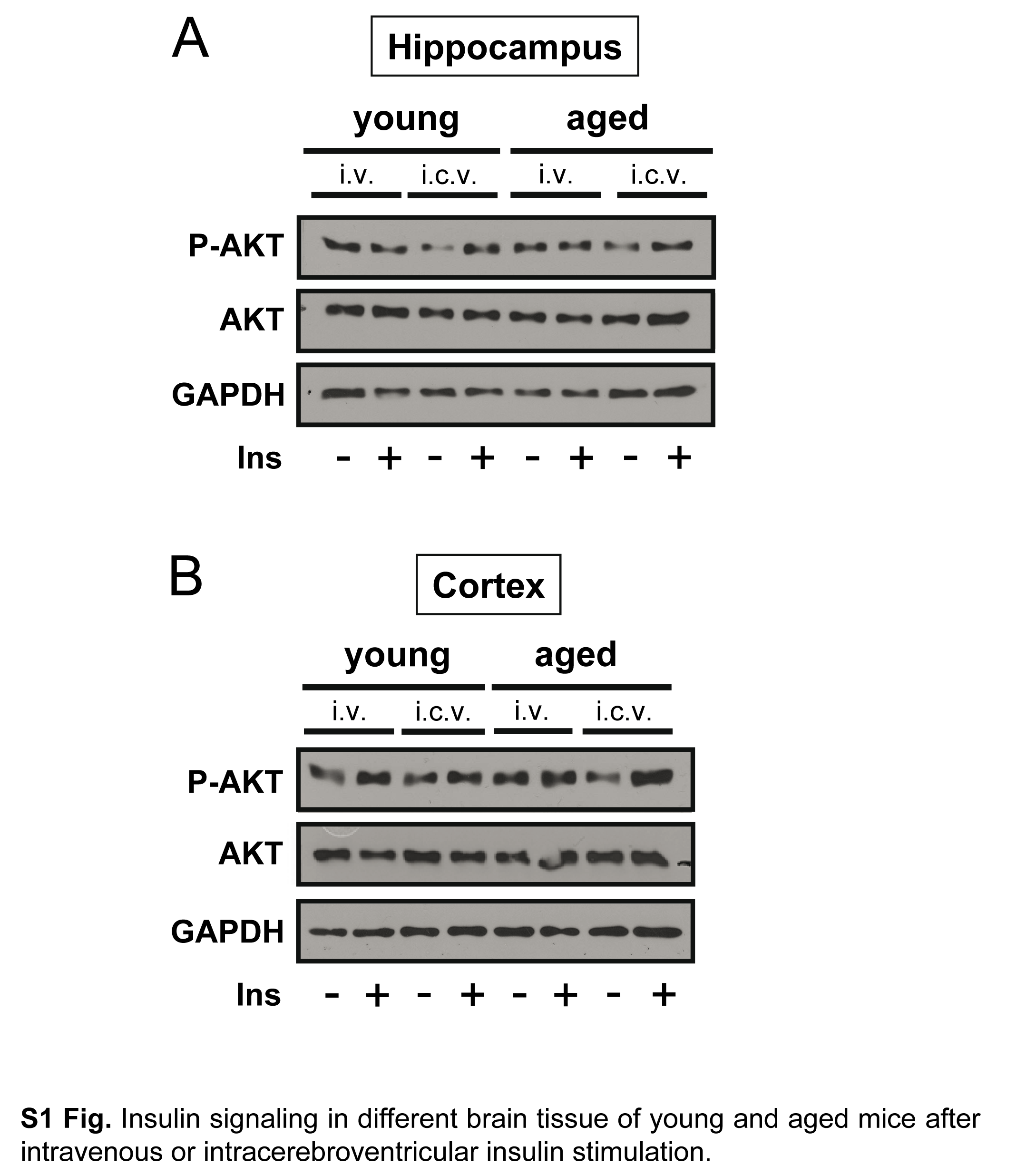

Supplement: S1 Fig — A-B: Western Blot analysis of phospho-AKT (Ser473) (P-AKT) and AKT in hippocampus (A) and cortex (B) after intravenous (i.v.) or intracerebroventricular (i.c.v.) human insulin or vehicle injection in overnight fasted young and aged animals. Parallel Western blots were run to detect unphosphorylated AKT. Ins, insulin. (TIF) [file pone.0126804.s001.tif]
